# Antimicrobial potential of bacteriocins in poultry and swine production

**DOI:** 10.1186/s13567-017-0425-6

**Published:** 2017-04-11

**Authors:** Amel Ben Lagha, Bruno Haas, Marcelo Gottschalk, Daniel Grenier

**Affiliations:** 1 Groupe de Recherche en Écologie Buccale (GREB), Faculté de médecine dentaire, Université Laval, Quebec City, QC Canada; 2 Groupe de Recherche sur les Maladies Infectieuses du Porc (GREMIP), Faculté de médecine vétérinaire, Université de Montréal, Saint-Hyacinthe, QC Canada; 3 Centre de Recherche en Infectiologie Porcine et Avicole (CRIPA), Fonds de Recherche du Québec-Nature et Technologies (FQRNT), Saint-Hyacinthe, QC Canada

## Abstract

The routine use of antibiotics in agriculture has contributed to an increase in drug-resistant bacterial pathogens in animals that can potentially be transmitted to humans. In 2000, the World Health Organization identified resistance to antibiotics as one of the most significant global threats to public health and recommended that the use of antibiotics as additives in animal feed be phased out or terminated, particularly those used to treat human infections. Research is currently being carried out to identify alternative antimicrobial compounds for use in animal production. A number of studies, mostly in vitro, have provided evidence indicating that bacteriocins, which are antimicrobial peptides of bacterial origin, may be promising alternatives to conventional antibiotics in poultry and swine production. This review provides an update on bacteriocins and their potential for use in the poultry and swine industries.

## Antibiotic use and resistance in poultry and swine production

Antibiotics are used in animal production for three main purposes: (1) as therapeutics to treat established infections, (2) as prophylactics/metaphylactics to prevent the development of bacterial diseases in clinically healthy animals, and (3) as growth promoters to improve feed conversion and body-weight gain. Despite the adoption of regulations banning the use of antibiotics for disease prevention and growth promotion by many countries, antibiotic-supplemented feeds can still be purchased without a veterinary prescription in a number of major animal-producing countries, including the United States, Canada, China, and Australia [1, 2]. These practices often expose bacteria to sublethal doses of antibiotics and consequently may favor the emergence of drug-resistant bacterial strains. It should be pointed out that a correctly provided therapeutic treatment also exerts antibiotic selective pressure that may promote antibiotic resistance.

Approximately 80% of all antibiotics sold or distributed in the United States in 2012 were for animal agriculture [3]. Sales of medically important antibiotics for use in food-producing animals are dominated by tetracyclines (67%), followed by penicillins (11%), macrolides (7%), sulfonamides (6%), aminoglycosides (8%), lincosamides (2%), and cephalosporins (<1%) [3]. A recent study from Germany revealed that tetracyclines were the most common antibiotics used in pigs, followed by beta-lactams and trimethoprim–sulfonamides [4]. From 2011 to 2014, a similar distribution in various food-producing animals, including pigs, poultry, and cattle, was observed in most European countries [5]. Antibiotics specifically used by the poultry and swine industries make up approximately 67% of global animal health antimicrobial needs [6].

While antibiotics are required to treat infections and maintain animal health, they are not essential for promoting animal growth. The use of antibiotics as antimicrobial growth promoters (AGP) is based on observations reported more than 50 years ago indicating that the addition of sub-therapeutic amounts of antibiotics to poultry and swine feeds had a significant growth-promoting effect on the animals, increasing weight gain and meat production [7]. Antibiotics benefit animal health at least in part by modulating the immune system and modifying the microflora of the gastrointestinal tract resulting in a reduction of the total bacterial load and suppression of pathogens [8]. One of the major benefits of AGP may be maintaining animal health in older facilities, where hygiene management is less efficient. However, the growth response to AGP appears to be much less important when animal nutrition, hygiene practices, as well as genetic potential and health status of animals are optimal. Indeed, recent studies (post-2000) have shown that productivity gains from AGP are lower than what was reported in earlier studies [7, 9, 10]. More specifically, Miller et al. [9] reported that the use of AGP in pork production increased the average daily weight by 0.5% and feed efficiency by 1.1%; that is much less that previously reported in the 1980s [10]. With respect to the broiler chicken industry in Denmark, the mortality rate, the average weight gain, and productivity from 1995 to 1999 were not affected by the ban of AGP [11]. In general, the current scientific evidence tends to suggest that it is possible for the swine and poultry industries with optimized production systems to maintain efficient production at cost-effective without using AGP [12].

Bacteria may become resistant to antibiotics through three major mechanisms: physiological adaptation, mutations, and transfer of resistance genes [13]. The extensive use of antibiotics in food-producing animals has contributed to an increase in drug-resistant animal pathogens that can potentially be transmitted to humans and negatively impact human health [14]. For instance, in the swine industry, most enterotoxigenic *Escherichia coli* strains are resistant to tetracyclines, aminoglycosides, trimethoprim–sulphonamides, and ampicillin [15]. The emergence of resistance to fluoroquinolones and colistin has also been reported [16, 17]. Moreover, *Streptococcus suis*, a swine pathogen and important zoonotic agent, is highly resistant to macrolides such as erythromycin, tetracyclines and, to a lesser extent, penicillin [18, 19]. Wang et al. recently discovered the *cfr* gene, which confers to *S. suis* a multi-resistance to five different classes of antibiotics (phenicols, oxazolidinones, lincosamides, pleuromutilins, and streptogramin A) [20]. Regarding the poultry industry, *E. coli* strains resistant to multiple antibiotics (tetracycline, ampicillin, streptomycin) are frequently isolated [21, 22]. Resistance genes have also been identified in *Salmonella* serovars, *Enterococcus* spp., and *Clostridium perfringens* recovered from feces and ceca of broiler chickens [23].

The transmission of resistant bacteria from animals to humans may occur through direct contact with animals or through the consumption of or contact with uncooked contaminated meat. Indirect transmission through the environmental pathways is also possible [24]. The transmission of resistance genes into human bacterial pathogens by horizontal gene transfer is also to consider although it remains relatively difficult to prove. It has been suggested that the increased bacterial resistance to certain antibiotics in both animals and humans is correlated with their addition to animal feed and their use in veterinary medicine. A typical example is fluoroquinolone resistance in *Campylobacter* species which has been associated with the use of these antibacterial agents in poultry [25]. In 2000, the World Health Organization (WHO) identified resistance to antimicrobial agents as one of the most significant global threats to public health and recommended that the use of antibiotics as additives in animal feeds should be phased out or terminated, particularly those used to treat human infections [26]. In order to attenuate this increase in antibacterial resistance, countries of the European Union banned the use of AGP in livestock in 2006. In the United States, progress in restricting AGP use in animal production has been relatively modest despite the fact that the Food and Drug Administration (FDA) adopted a voluntary policy in December 2013 recommending that producers stop the routine use of antibiotics and consult veterinarians before using them [27]. Pressure from consumers is likely to encourage the food-producing animal industry to eliminate the use of antibiotics in animal feeds. Few studies investigated whether reduction of antibiotic selection pressures through a ban is associated with a reduction in the occurrence of resistant bacteria. On the one hand, the decision to ban AGP in Denmark in 1986 resulted in a decrease in antibiotic-resistant bacteria in animals, food, and humans [28, 29]. On the other hand, in the USA, the ban of fluoroquinolones in chicken did not result in a declined incidence of fluoroquinolone-resistant *Campylobacter* strains [30]. However, it is of interest to mention that an increase in the use of antibiotics for a therapeutic purpose has been observed since the ban of AGP by the European Union countries, likely because of the producers’ expectations regarding performance enhancement and disease prevention [31].

Whenever and whatever antibiotics are used in animal production, it creates a selective pressure for the emergence of antibiotic-resistant bacteria. For this reason, there is a need for novel antibacterial compounds that can be used in animal production and for which bacteria do not easily develop resistance that may potentially be transmissible to human pathogens. In this regard, bacteriocins, which were discovered in 1925 by Gratia [32], are of great interest for controlling both animal and foodborne pathogens. This review provides an update on bacteriocins and their potential for use in the swine and poultry industries.

## Bacteriocins

### Generalities

Bacteriocins are ribosomally synthesized bactericidal or bacteriostatic peptides produced by certain bacteria. Although Gram-negative bacteria can produce bacteriocins, the vast majority of bacteriocins characterized so far are produced by Gram-positive bacteria [33]. BACTIBASE, an open-access database on bacteriocins, contains 177 bacteriocin sequences, of which 156 are from Gram positive bacteria and 18 are from Gram negative bacteria [33]. According to this database, the peptide length for bacteriocins produced by Gram positive bacteria ranges from 20 to 60 amino acid residues while bacteriocins from Gram negative bacteria have a wider range of lengths with the longest having 688 amino acid residues. The widespread occurrence of bacteriocins among bacterial species isolated from complex microbial communities suggests that these bacterial products may play a regulatory role in terms of population dynamics within bacterial ecosystems.

Bacteriocin production involves several genes implicated in the modification of amino acids, the export and regulation of the bacteriocin, as well as self-immunity [34]. These genes may be located on the chromosome or on a plasmid [34]. The producing bacteria usually synthesize self-immunity proteins that protect them from being killed by their own bacteriocins [35]. The immunity proteins protect cells by scavenging bacteriocins or by antagonistic competition for the bacteriocin receptor. While many bacteriocins have a narrow spectrum of activity, inhibiting the growth of similar or closely related bacterial species, others display antimicrobial activity against a broad array of genera [34]. Usually, the activity spectrum exerted by bacteriocins of Gram negative bacteria is narrower than those produced by Gram positive bacteria. Several bacteriocins have been shown to act in synergy with conventional antibiotics [36, 37], thus allowing to reduce bactericidal concentrations and decrease their undesirable side-effects. Interestingly, some bacteriocins produced by Gram positive bacteria have been found to be active against viruses [38, 39]. Bacteriocins, depending on their primary structure, may exert antibacterial activity through different modes of action on susceptible bacteria. Some act on the bacterial cell envelope causing cell lysis, while others are active once inside the cells, affecting gene expression and protein production [40].

As observed with the antibiotics, bacteria can develop resistance against bacteriocins principally through modifications of their cell envelope such as alterations in charge and thickness [41–44]. However, since bacteriocins have not been extensively tested in clinical setting yet, this aspect has been poorly investigated.

Bacteriocins possess a wide range of sizes, structures, modes of action, activity spectra, and target cell receptors. The classification of bacteriocins undergoes continuous modifications because of new developments regarding their structures and modes of action. According to the classification proposed by Heng and Tagg [45], they are divided into four classes. This classification may be considered universal since it includes bacteriocins from both Gram-positive and Gram-negative bacteria. The four classes include (I) lantibiotics, (II) non-lantibiotics or unmodified peptides, (III) high molecular mass peptides, and (IV) circular peptides (Table 1).

**Table 1:** **Classification of bacteriocins and their major features**

Class	Subclass	Example	Features
I (lantibiotic)	Ia (linear)	Nisin A	• MW < 5 kDa • Linear peptide • Presence of modified amino acids (lanthionine, methylanthionine) • Heat-stable
	Ib (globular)	Suicin 3908	
	Ic (multi-components)	Lacticin 3147	
II (non-lantibiotic)	IIa (pediocin-like)	Pediocin PA-1	• MW < 10 kDa • Linear peptide • Absence of modified amino acids • Heat-stable
	IIb (miscellaneous)^a^	Aureocin A53	
	IIc (multi-components)	Lactococcin G	
III	IIIa (bacteriolytic)	Lysostaphin	• MW > 25 kDa • Linear peptide • Heat-sensitive
	IIIb (non-lytic)	Helveticin J	
IV	None	Enterocin AS-48	• MW < 8 kDa • Cyclic peptide

^a^This subclass includes bacteriocins with distinct features.

### Classification

#### Class I (lantibiotics)

Class I bacteriocins, or lantibiotics, are low molecular mass peptides (2–5 kDa) produced by Gram-positive bacteria [46]. They are thermostable and resistant to extreme pHs and certain proteases. The main feature that differentiates them from other bacteriocins is the presence of atypical post-translationally, enzymatically modified amino acids such as lanthionine, methyllanthionine, dehydroalanine, and dehydrobutyrine [46]. The most widely recognized representative of this class is nisin, which is produced by *Lactococcus lactis* subst. *lactis*. Lantibiotics have a dual mode of action against susceptible bacteria [40]. They can bind to lipid II, the major transporter of peptidoglycan subunits, interfering with cell wall synthesis. In addition, they can also use lipid II as a docking molecule, leading to pore formation and cell death. Because of this mode of action, high resistant mutants to lantibiotics cannot develop. Lantibiotics can be sub-divided into three categories based on their structures and functional properties: linear (type a), globular (type b), and multi-component (type c). The lantibiotic biosynthesis machinery is encoded by gene clusters that typically include a structural gene for a pre-lantibiotic peptide as well as genes required for the modification of amino acids, export, regulation, and self-immunity [46]. More specifically, the structural gene encodes a prepeptide containing a leader sequence at the N-terminus, which is eventually cleaved, and a propeptide at the C-terminus in which many or all of the serine and threonine residues are modified.

#### Class II (non-lantibiotics or unmodified peptides)

Class II bacteriocins (non-lantibiotics) are thermostable, small (<10 kDa) peptides with an amphiphilic helical structure that do not contain modified amino acid residues [47]. Generally, the class II bacteriocins target the lipid II as the lantibiotics or bind to the pore-forming receptor mannose phosphotransferase system [40]. This class is divided into three sub-classes (IIa, IIb, IIc). Class IIa bacteriocins (pediocin-like) include peptides that have the YGNGVXC consensus sequence at the N-terminus and that display a strong inhibitory activity against the food pathogen *Listeria monocytogenes* [47]. Class IIb bacteriocins contain heterogeneous linear peptides unrelated to pediocin. Lastly, class IIc bacteriocins comprise bacteriocins that require two unmodified peptides in about equal quantities to exert their antibacterial activity. When tested individually, these bacteriocins display low if any activity. Each peptide pair, that differs in their amino acid sequence, is encoded by two genes located in the same operon.

#### Class III (high molecular mass peptides)

Class III bacteriocins, which have been poorly studied, include large, thermolabile peptides (>10 kDa) and are divided into two sub-classes: bacteriolytic (IIIa) and non-lytic peptides (IIIb) [47]. The mode of action of bacteriolytic peptides is different from that of other bacteriocins as they catalyze peptidoglycan hydrolysis resulting in lysis and death of the target cells. Non-lytic peptides, as their name implies, do not cause cell lysis. Depending on the bacteriocin, different mechanisms may be used such as membrane leakage of small molecules, inhibition of sugar uptake, and inhibition of DNA synthesis [48].

#### Class IV (circular peptides)

Lastly, class IV bacteriocins are post-translationally modified circular peptides that possess a covalent bond between the N- and C-terminals. The circular bacteriocins that have been identified so far are all produced by Gram positive bacteria [49]. The circular bacteriocins possess a wide activity spectrum and exhibit high resistance to heat, extreme pHs, and proteolytic enzymes [49]. These bacteriocins generally exert their antibacterial action by disruption of the membrane integrity [50].

### General uses of bacteriocins

Most studies on bacteriocins produced by food-grade lactic acid bacteria have investigated the food preservation potential of the bacteriocins because of their impressive in vitro and in situ efficacy against foodborne pathogens [51, 52]. In this context, several strategies have been proposed: (1) the addition of a purified or semi-purified bacteriocin as a food preservative, (2) the use of a product previously fermented with a bacteriocin-producing bacterium as an ingredient/supplement in the food preparation process, and (3) the inoculation of food with the bacteriocin-producing strain.

Nisin A, which is produced by *L. lactis* subsp. *lactis*, is the most studied bacteriocin and was approved in 1988 as a generally recognized as safe (GRAS) by the FDA due to its low toxicity in humans [53]. It is a lantibiotic (type a; 34 amino acid residues) that is commercially used as a food preservative (E234, Nisaplin^®^), especially in dairy products, in more than 50 countries, including the United States and several member countries of the European Union [54]. Although the main application of nisin is as a natural food bio-preservative, its recognized potential has been extended to the biomedical field, including the prevention/treatment of infectious diseases [55]. A nisin-based formulation (Wipe Out^®^ Dairy Wipes; Immucell, USA) has also been shown to be effective for treating mastitis caused by *Staphylococcus aureus*, *Streptococcus uberis*, and *Streptococcus dysgalactiae* in lactating dairy cows [56, 57].

Given the increasing concern about the acquisition of antibiotic resistance by pathogens, bacteriocins are being given serious consideration as a viable strategy to replace conventional antibiotics or to potentiate their effects against pathogens [58]. Unlike antibiotics, most bacteriocins are relatively specific and can thus be used to target particular pathogenic or non-beneficial bacteria without affecting the indigenous microflora. New bacteriocins with promising in vitro antimicrobial profiles and in vivo effectiveness are currently being identified. The use of bacteriocins as alternatives or adjuncts to help alleviate antibiotic overuse and resistance has become a very real possibility. In vivo trials with animal models have shown that lantibiotics can successfully prevent or treat infections. For example, nisin F (*L. lactis*) prevents respiratory infections by *S. aureus* in a rat model [59], while mersacidin, a lantibiotic produced by *Bacillus* spp., can eradicate MRSA colonization in a mouse rhinitis model [60]. Lastly, lacticin (*L. lactis*) has been reported to successfully control the systemic spread of *S. aureus* in a mouse model [61]. Interestingly, bacteriocins are considered to be natural antimicrobials that are nontoxic for eukaryotic cells [62].

Additional properties have recently been associated with lantibiotics [58]. Chopra et al. [63] reported that a bacteriocin produced by *Bacillus sonorensis* can prevent adherence and biofilm formation by food spoilage bacteria. Several bacteriocins produced by Gram-positive bacteria have also been reported to possess anti-cancer properties [64].

## Bacteriocins in poultry and swine production

### Poultry pathogens

The poultry industry has become an important economic activity in many countries. The impact of diseases on poultry production is one of the major factors that limit the success of this industry [65]. The high incidence of bacterial infections combined with the increase in drug-resistant pathogens is pushing the poultry industry to develop novel antimicrobial strategies. The potential of using bacteriocins to inhibit bacterial pathogens that affect the poultry industry and cause important economic losses is currently being investigated by several groups. Most studies on this aspect have focused on *C. perfringens* and *E. coli*, two major pathogens in the poultry industry.

The Gram-positive spore-forming anaerobic bacterium *C. perfringens*, which colonizes the intestinal tract of chickens, is the causative agent of necrotic enteritis [66]. Given the production losses and animal mortality, *C. perfringens* is considered to be the main disease of concern for poultry producers worldwide [66]. Since this bacterial species is also a foodborne pathogen associated with poultry, antimicrobial agents that can inhibit or kill it may also contribute to preventing potential food safety problems. Timbermont et al. [67] purified and characterized a bacteriocin (perfrin) produced by a strain of *C. perfringens* isolated from a chicken with necrotic enteritis. The 11.5-kDa bacteriocin is effective against other strains of type A *C. perfringens*. Given that the bacteriocin is only produced by strains expressing the NetB toxin and that the bacteriocin-producing strain is not susceptible to perfrin, the authors suggested that the bacteriocin likely contributes to the pathogenesis of necrotic enteritis by enabling the producing strain to outcompete other *C. perfringens* strains in the gut rather than by acting as a peptide with therapeutic potential [67]. Han et al. [68] conducted a search for bacteria with antagonistic activity against *C. perfringens* and isolated six *Enterococcus faecalis* strains from pig feces that inhibit *C. perfringens*. Although they did not purify the bacteriocin-like inhibitory substances, they suggested that these bacteria or their antibacterial compounds may be useful alternatives to antibiotics in the poultry industry. Several *Bacillus* spp., including *Bacillus cereus* [69], *Bacillus subtilis* [70, 71], *Bacillus pumilus* [72], and *Bacillus licheniformis* [72] have been reported to exert antagonism against *C. perfringens* through the production of bacteriocins. Jayaraman et al. [73] investigated the effect of a bacteriocin-producing *B. subtilis* strain, isolated from healthy chicken gut and used as a dietary supplement, on intestinal health and gut integrity in broiler chickens infected with *C. perfringens*. It was shown that supplementation with *B. subtilis* reduces intestinal lesion score and significantly lowers the intestinal *C. perfringens* counts compared with the infected control group [73]. Grilli et al. [74] reported that pediocin A, a bacteriocin produced by *Pediococcus pentosaceus*, was highly active against *C. perfringens* in an in vitro assay. They also showed that a partially purified fraction of pediocin A, alone or in association with the producer strain, significantly improves the growth performance of broiler chickens challenged with *C. perfringens* [73]. A recent study by Udompijitkul et al. [75] showed that nisin exerts strong antimicrobial activity against *C. perfringens* in vitro. In addition, Jozefiak et al. [76] reported that dietarynisin significantly and dose-dependently increases feed conversion by and the growth performance of broiler chickens. Like salinomycin, a widely used ionophore coccidiostat, nisin exerts a modulating effect on the microbiota of the gastrointestinal tract by decreasing counts of *Bacteroides* and *Enterobacteriaceae* [76]. It thus represents an effective dietary supplement for broiler chickens. Similar effects were reported for other bacteriocins, including diversion produced by *Carnobacterium divergens* [77] and albusin B produced by *Ruminococcus albus* [78].

*Escherichia coli* is a Gram-negative bacterium that is a normal member of the gastrointestinal microflora of poultry. Some strains can, however, cause severe diseases, commonly referred to as colibacillosis in chickens [79]. Systemic infections develop when large numbers of avian pathogenic *E. coli* gain access to the bloodstream from the respiratory or intestinal tract, especially if the host is stressed or their immune system is compromised [79]. Torshizi et al. [80] screened lactic acid bacteria isolated from chicken intestinal samples and found two isolates (*Lactobacillus fermentum* and *Lactobacillus rhamnosus*) with the capacity to inhibit the growth of *E. coli* in vitro. The nature of the inhibitory substances produced by these isolates suggests that they may be bacteriocins. Ogunbanwo et al. [81] investigated the potential therapeutic efficacy of bacteriocin and bacteriocin-producing *Lactobacillus plantarum* strain in an experimental *E. coli* infection of broiler chickens. They found that when chickens infected with *E. coli* are treated with bacteriocin alone or bacteriocin-producing *L. plantarum*, their health status is comparable to that of uninfected control chickens [81].

### Swine pathogens

The swine industry has experienced remarkable growth over the past 20 years. In addition to be linked to the increased demand, this trend is also related to the industrialization of processes, mass rearing, and increased efficiency of slaughterhouses, which have reduced production costs. In 2012, the swine industry dominated the world meat market with 36.3% of total production [82]. Between 2010 and 2012, the industry produced nearly 100 million tons of meat per year on average, half in China [82]. Increasing the capacity of farms and slaughterhouses has concentrated a large number of animals in confined spaces, making it harder to control bacterial and viral pathogens.

Post-weaning diarrhea is responsible for major economic losses in the swine industry [83]. Enterotoxigenic *E. coli* (ETEC) is the major cause of this enteric disease in pigs, being responsible for approximately 50% of piglet mortality [83]. Al Atya et al. [84] showed that combining colistin with bacteriocins (nisin, enterocin) from lactic acid bacteria enhances its in vitro antibacterial activity against planktonic and biofilm cultures of *E. coli*. They suggested that colistin disrupts the outer membrane of *E. coli* by acting on lipopolysaccharide, opening the way for the subsequent action of the bacteriocins [84]. Colicins, a class of bacteriocins produced by and active against *E. coli*, have been investigated as a possible alternative to antibiotics in swine production [85]. Colicin E1 inhibits the growth of *E. coli* strains that cause post-weaning diarrhea and edema disease in pigs, as shown in vitro [86]. Furthermore, Cutler et al. [87] showed that the addition of colicin E1 to the diet of piglets decreases the incidence and severity of experimental post-weaning diarrhea induced by an enterotoxigenic strain of *E. coli* and improved the growth performance of piglets. They also used a gene expression analysis (IL-β, TNF-β) to show that the inflammatory response occurring in ileal tissues leading to diarrhea was decreased. These promising results indicate that the use of colicins may have a positive impact on food safety since enterotoxigenic *E. coli* is considered to be an important foodborne pathogen.

*Haemophilus parasuis* causes Glässer’s disease in young pigs [88]. This systemic disease is characterized by polyserositis and fibrinopurulent polyarthritis. Teixeira et al. [89] reported the isolation of a low molecular mass bacteriocin from a reference strain of *B. subtilis* subsp. *spizezinii* (ATCC 6633). The bacteriocin was highly effective against approximately half the *H. parasuis* strains tested and may be a potential alternative to antibiotics for controlling infections caused by this pathogen.

*Streptococcus suis*, a common inhabitant of the tonsils of healthy pigs, is a major swine pathogen that has been associated with severe infections such as meningitis, arthritis, endocarditis, pneumonia, and septicemia [90]. It is one of the main bacterial pathogens responsible for major economic losses in the swine industry worldwide. In addition, this Gram positive bacterium is recognized as an emerging zoonotic agent for humans exposed to sick pigs or their by-products and has caused major outbreaks in Asia [91]. The nisin-producing strain *L. lactis* subsp. *lactis* ATCC 11404 exerts antagonistic activity toward *S. suis*, suggesting that this bacterial species may represent a probiotic of interest for the control of *S. suis* infections [92]. Moreover, all the *S. suis* isolates tested were susceptible to purified nisin, with MIC values ranging from 1.25 to 5 µg/mL [92]. When nisin was combined with conventional antibiotics such as amoxicillin and ceftiofur, which are commonly used to treat *S. suis* infections, strong synergistic effects were obtained [92]. These in vitro results provide support for the potential of nisin, a lantibiotic licensed as a food preservative, for preventing swine infections caused by *S. suis*. The purification and characterization of three lantibiotics, named suicins 90–1330, 3908, and 65, produced by three distinct strains of *S. suis* (serotype 2) have recently been reported [93–95]. Interestingly, all three producing strains were non-virulent in mouse/pig infection models, and two of them were isolated from healthy carrier pigs [93–95]. The distribution of suicin gene clusters in *S. suis* serotype 2 belonging to sequence type (ST) 25 and ST28, the two dominant STs in North America, was recently investigated [96]. The gene clusters encoding suicin 65 (mostly in ST25 strains) and, to a lesser extent, suicin 90–1330 (exclusively in ST28 strains) are the most prevalent. Since all three suicins are bactericidal for highly virulent ST1 *S. suis* strains, which are mainly found in Eurasia, the use of the semi-purified bacteriocin preparations or the bacteriocin-producing strain may represent a valuable strategy for controlling *S. suis* infections and for reducing antibiotic use in the swine industry.

In 2012, Riboulet-Bisson et al. [97] evaluated the impact of *Lactobacillus salivarius* administration and, more specifically, the effect of bacteriocin production by this bacterium on the intestinal microbiota of healthy pigs. *L. salivarius* strain UCC118 is a well-known probiotic bacterium of human origin that produces a broad-spectrum class IIb bacteriocin [98]. Administering the bacteriocin-producing *L. salivarius* resulted in the modulation of the Gram-negative bacterial population of the intestinal microflora, decreasing the levels of *Bacteroidetes* and *Spirochaetes* [97]. Such an effect was not observed with a mutant lacking bacteriocin production. Although members of these two phyla are mostly commensals, under certain conditions they may become opportunistic pathogens in humans and animals. For example, *Treponema* spp. and *Bacteroides* spp. can cause colitis [99] and diarrhea [100], respectively.

Pediocin is a broad-spectrum class IIa bacteriocin produced by *P. pentosaceus* [101]. Casadei et al. [102] investigated the in vitro effects of pediocin A, on microbial metabolism in the small and large intestines of pigs. While pediocin A had no effect on the fermentation parameters of the small intestine, it significantly reduced the growth of harmful bacteria, including clostridia and coliforms, and increased the metabolic activity of cellulolytic bacteria [102]. Based on these observations, the authors suggested that pediocin A could be an alternative to replace AGP and to improve the production of farm animals.

### Foodborne pathogens

Poultry and poultry products (particularly eggs) are considered to be a major source of human infections, being responsible for approximately 50% of foodborne disease outbreaks [103]. The use of bacteriocins and bacteriocin-producing bacteria may be a viable strategy for reducing the colonization of the gastrointestinal tract of poultry by foodborne pathogens, including *Campylobacter jejuni* and *Salmonella enterica*. The prevalence of *Campylobacter* colonization, particularly of *C. jejuni*, in broiler flocks is highly variable but can reach 60–80% of flocks of slaughter age in Europe and the United States [104]. While *C. jejuni* is not considered to be a poultry pathogen, it is a foodborne and human pathogen of primary importance [105]. *C. jejuni* infections in humans are a leading cause of diarrheal disease and foodborne gastroenteritis worldwide [105]. *C. jejuni* infections (campylobacteriosis) are mostly associated with the consumption of undercooked poultry products [106]. The use of bacteriocins active against *C. jejuni* may be a promising strategy to improve food safety and protect public health [107, 108]. Over the past ten years, several studies have reported that numerous bacterial species in poultry, including *L. salivarius* [109, 110], *Bacillus circulans* [111] *Paenibacillus polymyxa* [111], *Enterococcus faecium* [112, 113], *Carnobacterium divergens* [114], *Leuconostoc mesenteroides* [115], and *Lactobacillus sakei* [116] produce bacteriocins that are active against *C. jejuni*. Interestingly, some of these bacteriocins possess desirable properties for in vivo applications, including heat and low pH tolerance, simple production and extraction processes, and no toxicity toward eukaryotic cells. The oral administration of semi-purified bacteriocins to effectively control foodborne pathogens can be easily achieved by incorporating them into feed or drinking water. Stern et al. [117] showed that incorporating purified bacteriocin B602 (secreted by *P. polymyxa*) encapsulated in polyvinylpyrrolidone in chicken feed significantly reduces the intestinal levels and the frequency of chicken colonization by *C. jejuni*. B602-supplemented feeds have also been shown to reduce *Campylobacter* colonization to undetectable levels in turkeys [118]. Bacteriocin OR-7, which is produced by a *L. salivarius* strain isolated from the cecum of a commercial broiler, also inhibits *C. jejuni* in vitro [119]. Polyvinylpyrrolidone-encapsulated bacteriocin OR-7 added to chicken feeds reduces *C. jejuni* colonization at least one million-fold compared with control birds [119]. Line et al. [120] showed that enterocin E-760, which was isolated from *Enterococcus* sp. NRRL B-30745, reduces the colonization of naturally acquired *Campylobacter* species in market age broiler chickens when administered in feeds [120], More specifically, the administration of enterocin E-760 was associated with an impressive 8-log reduction in *Campylobacter* counts in broiler chickens [120].

*Salmonella* is a major foodborne pathogen responsible for more than a million illnesses annually in the United States alone due to the consumption of animal products: poultry, poultry products, meat and dairy [121]. The poultry industry has also investigated the use of bacteriocins and/or bacteriocin-producing bacteria for their ability to control *Salmonella* [122–124]. Several studies have indicated that such probiotics, either inoculated orally into chickens or incorporated into feed, inhibit the growth of enteric pathogens such as *Salmonella*, likely by competitive exclusion or by the production of antimicrobial metabolites [122–124]. The administration of bacteriocin-producing bacteria can have a direct effect on reducing existing populations of *Salmonella*, while long-term colonization with bacteriocin-producing bacteria may prevent the reintroduction of *Salmonella* [122]. The bacteriocin albusin B, which is produced by *Ruminococcus albus* 7, has been reported to increase intestinal nutrient absorption, elevate fecal *Lactobacillus* counts, and decrease *Salmonella* loads, thereby improving the growth performance of broiler chickens [78]. Van Winsen et al. showed that *L. plantarum* does not display antimicrobial activity against *Salmonella*, but its addition to swine feeds resulted in a competition phenomenon that inhibits *Salmonella* growth and allows *L. plantarum* to dominate the intestinal flora [125]. The administration of bacteriocin-producing bacteria rather than the bacteriocins themselves might be a more cost-effective approach, but significant progress in developing suitable producer strains will have to be made before such an approach becomes feasible.

## Conclusions

**Figure 1:**
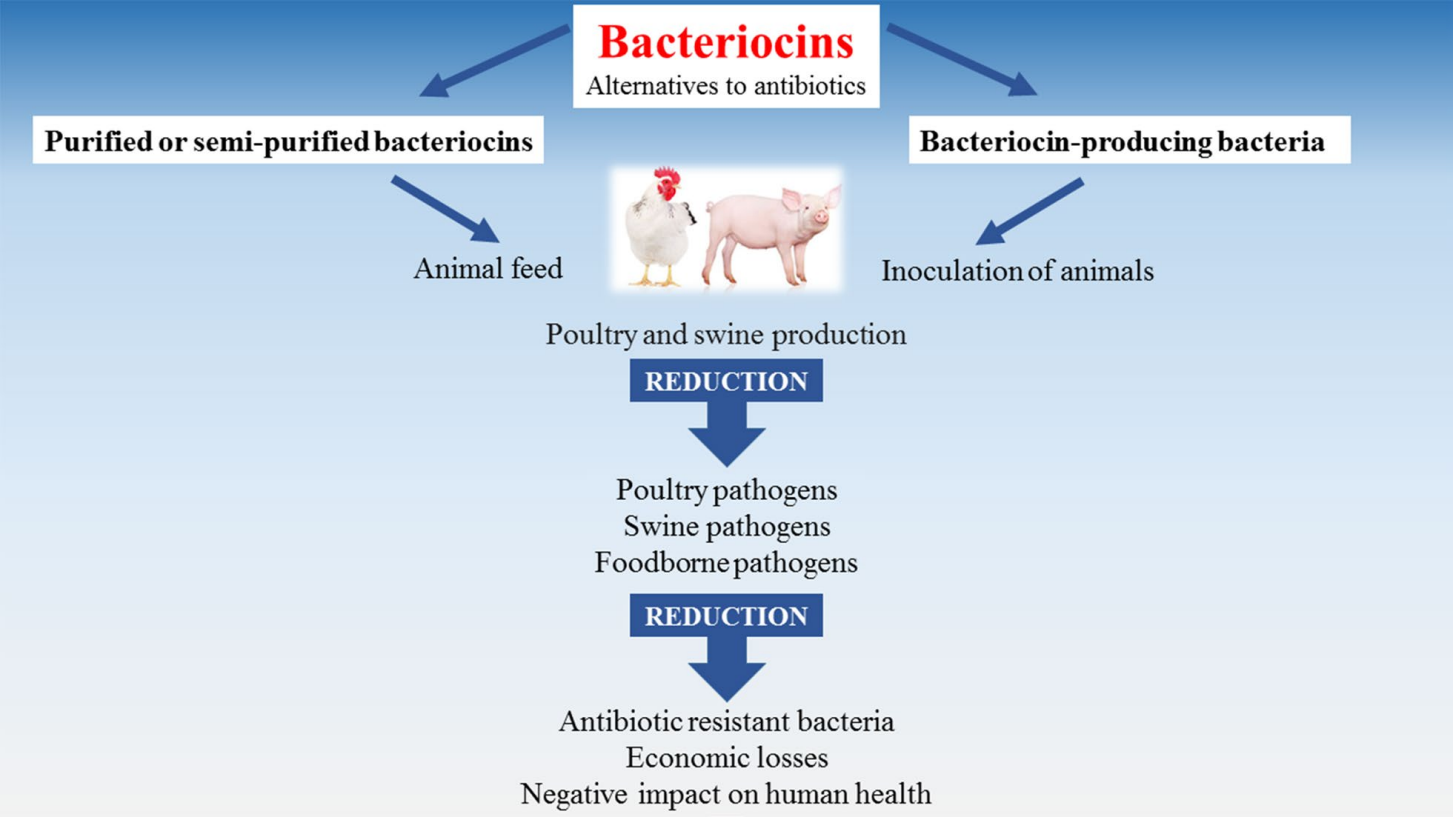
**Potential benefits of bacteriocins and bacteriocin-producing bacteria in poultry and swine production.** Bacteriocins, either purified or semi-purified, may be directly added to animal feed as anti-infective additives to protect animals. Alternatively, the bacteriocin-producing bacteria may be used as probiotics and inoculated into animals to allow colonization and protection against bacterial pathogens. Both procedures may reduce the amounts of animal pathogens as well as foodborne pathogens. As a consequence, this may decrease the emergence of antibiotic resistant bacteria, the economic losses and the negative impacts on human health.

As more countries develop antibiotic-limiting policies, the need for alternative antimicrobials will likely become the main driving force behind the identification of novel bacteriocins and the testing of existing ones. The use of semi-purified bacteriocins or bacteriocin-producing bacteria in animal production is a field with enormous research and commercialization potential. Bacteriocins hold great promise for the prevention and/or treatment of bacterial diseases and may eventually be employed as alternatives to antibiotics (Figure 1).

Bacteriocins not only represent alternatives to AGP but are also considered as promising therapeutic agents for animal disease prevention, control, and treatments. Several clinical and pre-clinical trials have explored the potential of purified bacteriocins as effective antimicrobial therapeutics or prophylactics and have given promising results [81, 118]. A number of issues remain to be addressed, however, including the production cost, dosage, timing, and in vivo activity of each bacteriocin. Since bacteriocins can be digested in the gastrointestinal tract, the administration of bacteriocin-producing bacteria rather than the bacteriocins themselves may be a more effective approach. In fact, the use of bacteriocin producers as probiotics may be cost-effective, and could target specific pathogens without affecting beneficial bacteria. A combination of multiple strains producing different bacteriocins might more efficiently target pathogenic bacteria. However, several conditions must be met, including the successful colonization of the digestive tract by the bacteriocin-producing bacteria and the actual production of bacteriocins in this environment.

Bacteriocins can also be used to decrease the numbers of potentially pathogenic bacteria in waste water and manure in order to limit their transmission to humans. Lauková et al. [126] evaluated the effectiveness of bacteriocin CBE V24, which is produced by *E. faecalis* V24, in reducing the numbers of potential human pathogenic bacteria found in cattle dung water and manure. They suggested that bacteriocins such as CBE V24 could be used to better manage animal excrement and waste water without resorting to antibiotics [126]. These applications could be easily transferred to the poultry and swine industries. However, further research is required to confirm this possibility.
